# The role of intercellular communication in diabetic nephropathy

**DOI:** 10.3389/fimmu.2024.1423784

**Published:** 2024-08-22

**Authors:** Bihan Wang, Yonghong Xiong, Xinqi Deng, Yunhao Wang, Siyuan Gong, Songyuan Yang, Baichuan Yang, Yuhang Yang, Yan Leng, Wenyuan Li, Wei Li

**Affiliations:** ^1^ Department of Anesthesiology, Renmin Hospital of Wuhan University, Wuhan, China; ^2^ Department of Urology, Renmin Hospital of Wuhan University, Wuhan, China; ^3^ The First Clinical College of Wuhan University, Wuhan, China

**Keywords:** diabetic nephropathy, intercellular communication, tunnel nanotube, exosome, filopodial tip vesicle, fibrogenic niche, single-cell RNA sequencing

## Abstract

Diabetic nephropathy, a common and severe complication of diabetes, is the leading cause of end-stage renal disease, ultimately leading to renal failure and significantly affecting the prognosis and lives of diabetics worldwide. However, the complexity of its developmental mechanisms makes treating diabetic nephropathy a challenging task, necessitating the search for improved therapeutic targets. Intercellular communication underlies the direct and indirect influence and interaction among various cells within a tissue. Recently, studies have shown that beyond traditional communication methods, tunnel nanotubes, exosomes, filopodial tip vesicles, and the fibrogenic niche can influence pathophysiological changes in diabetic nephropathy by disrupting intercellular communication. Therefore, this paper aims to review the varied roles of intercellular communication in diabetic nephropathy, focusing on recent advances in this area.

## 1 Introduction

Diabetic nephropathy (DN),characterized by renal interstitial inflammation and fibrosis, is the key factor of end-stage renal disease (ESRD) (1). The global rate of ESRD among diabetics increased from 19.0% in 2000 to 29.7% in 2015 the rate of diabetes-induced ESRD events has increased from 22.1% to 31.3% (2). Another study showed that 60.3% of patients with stage 4 chronic kidney disease (CKD) and DN rapidly progressed to ESRD or died after treatments, with deaths accounting for 10.9% of patients (3). This indicates an increasing number of people suffer from the DN’s devastating consequences, and that despite the constant updating of therapeutic measures, these measures have not entirely stopped the progression of DN. Consequently, a significant number of patients continue to suffer from ESRD. Therefore, identifying new therapeutic targets and strategies to improve DN’s prognosis is a hot topic of research and a current challenge.

**Figure 1:**
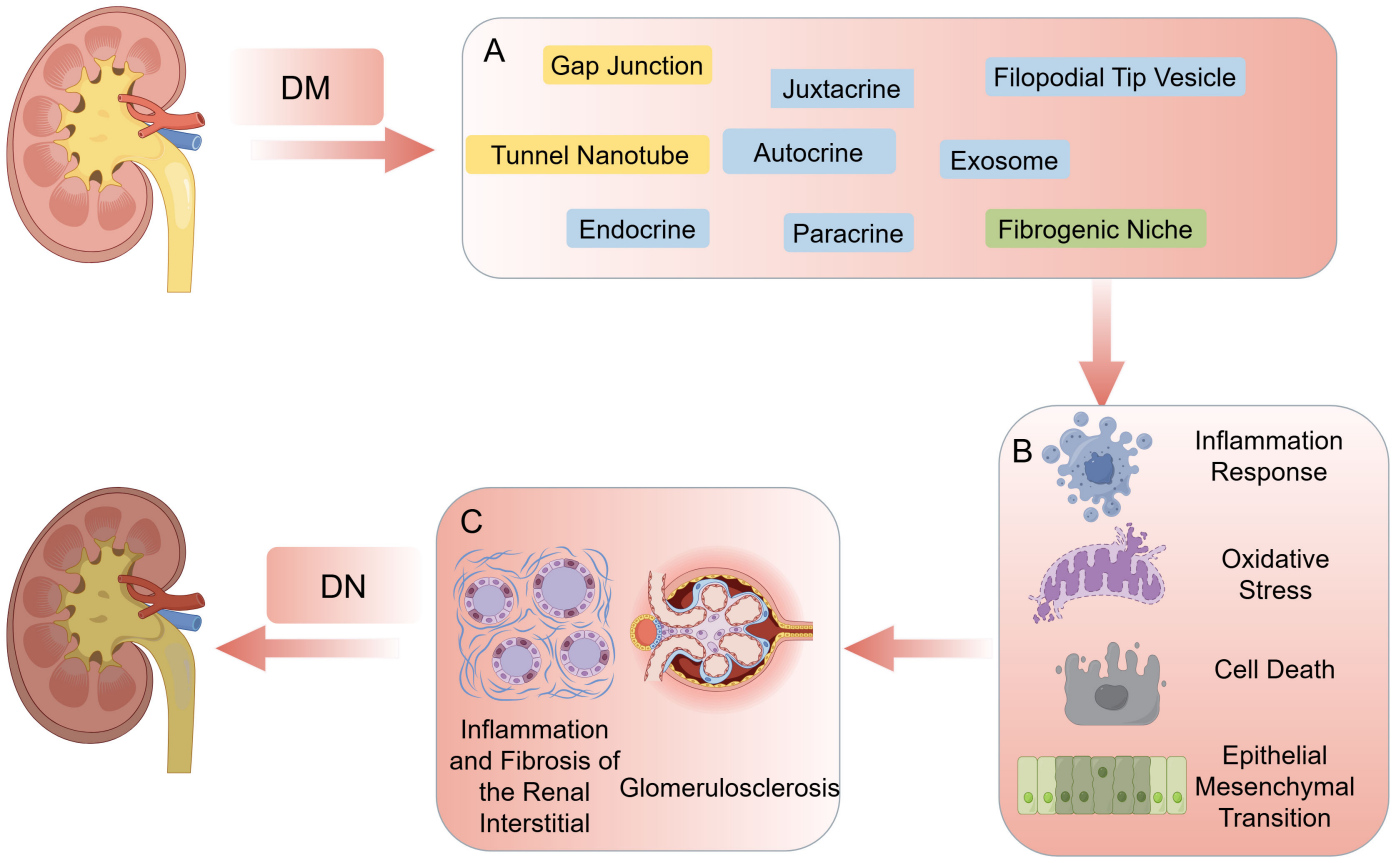
**(A)** The main ways in cell-cell communication. **(B)** Cell responses including inflammation response, oxidative stress, cell death, epithelial mesenchymal transition. **(C)** The consequence of high-glucose environment: glomerulosclerosis, inflammation and fibrosis of the renal interstitial. By figdraw.

In recent years, research on the mechanism of pathogenesis of DN has advanced, involving several complex biological processes. Currently, there are four main pathological mechanisms have been identified (4–6): 1) Hyperglycemia increases the intracapillary pressure in glomerular capillaries, leading to glomerular hyperfiltration and hyperperfusion, which in turn thickens of the glomerular basement membrane and accumulates extracellular matrix (ECM) in the tethered zone. 2) Hyperglycemia promotes the formation of glycosylation end products, binding to renal cell receptors and activating signaling pathways including protein kinase C, transforming growth factor-β, and nuclear factor-κB, leading to oxidative stress, inflammatory responses, apoptosis, and fibrosis in renal cells. 3) Hyperglycemia activates polyol metabolic pathways, leading to the accumulation of sorbitol and fructose in renal cells, causing cellular edema, increased osmolality, glycosylation of ECM, and impairment of cellular function and metabolism. 4) Hyperglycemia stimulates renin-angiotensin-aldosterone system activation and increase angiotensin II production, leading to constriction of renal vasculature, glomerulosclerosis, and increase risk of interstitial fibrosis. Simultaneously, multiple stimuli in diabetes induce cytokine and chemokine production, and peptide signaling molecules promote autocrine, paracrine, and proximal signaling. Meanwhile, in the kidney, blood-derived cells as well as a variety of intrinsic renal cells (glomerular, endothelial, tubular, and mesangial cells) synthesizing inflammatory cytokines, leading to immune and inflammatory responses in renal tissue. These pathogenic mechanisms manifest histologically as thickening of the glomerular and thylakoid membranes, and podocyte loss, leading to glomerulosclerosis, renal interstitial inflammation and fibrosis, and progressive renal function decline (**Figures 1B, C**) (7).

Various treatments are available to address DN, primarily focusing on the following mechanisms:1) Blood glucose control: Managing blood glucose levels with hypoglycemic drugs or insulin to minimize renal damage caused by hyperglycemia. 2) Blood pressure control: Using ACE inhibitors (ACEIs) or angiotensin II receptor blockers (ARBs) to reduce glomerular hyperperfusion and hyperfiltration, thereby decreasing the thickening of glomerular basement membranes and ECM accumulation. 3) Anti-inflammatory and antioxidant treatments: Addressing oxidative stress and inflammation caused by glycosylation end-products with anti-inflammatory drugs and antioxidants, such as SGLT2 inhibitors and GLP-1 receptor agonists, to reduce renal cell injury. 4) Blockade of the polyol metabolic pathway: Reducing the accumulation of sorbitol and fructose through pharmacological interventions, such as aldose reductase inhibitors, to alleviate cellular edema and osmotic pressure increase. 5) Regulation of the renin-angiotensin-aldosterone system: Using ACEIs or ARBs to reduce the production of angiotensin II, thereby lowering the risk of renal vasoconstriction and glomerulosclerosis. Additionally, nutritional management and maintaining a healthy lifestyle are crucial throughout the treatment process (8).

The pathophysiology of DN is complex and multifaceted. Although it is well recognized that chronic hyperglycemia is a primary factor in the development of DN, strict glycemic control does not always slow or prevent disease progression. Other mechanisms involved in DN, including inflammation, fibrosis, and oxidative stress, also play crucial roles in the progression of the disease (9). Given the complexity of these mechanisms, it is important to consider whether there are interactions between different pathogenic mechanisms and how cellular communication among various participating cells occurs, in order to find ways to maintain or disrupt balance to delay DN progression. Previous literature has suggested that (10) cross-talk between different types of kidney cells mediates changes in the renal microenvironment. Additionally, emerging evidence indicates that abnormal communication between renal cells contributes to the pathogenesis of DN (11). Thus, it is clear that the mechanisms of cellular communication involved in the progression of diabetic kidney disease are highly complex. A thorough understanding of these mechanisms will help identify potential therapeutic targets.

In addition, single-cell sequencing studies (12) have illuminated the intricate interplay among renal parenchymal cells, resident immune cells, and infiltrating immune cells during the development of DN, revealing a dynamic network of interactions orchestrated by multiple signaling molecules. Such findings suggests that targeting intercellular communication pathways could potentially delay or even impede the progression of DN from various perspectives. Traditionally, Intercellular communication includes direct communication (gap junctions, juxtacrine interactions) and indirect communication, represented by chemical signaling (13). However, recent discoveries have expanded this understanding to include the roles of tunnel nanotubes (TNTs), exosomes and filopodial tip vesicles (FTVs) in DN-related intercellular communication, offering new avenues for intervention (9). Moreover, studies have highlighted the significance of the ECM, which serves as a scaffold for intercellular communication and contains various matrix proteins that influence renal intercellular interactions. The ECM appears to play a crucial role in slowing the progression of renal fibrosis(RF) in DN, forming a complex network with cells known as the fibrogenic niche. The ensuing discussion will delve into recent advancements in understanding intercellular communication in DN and its potential implications for therapeutic interventions (**Figure 1A**).

## 2 Direct communication

**Figure 2:**
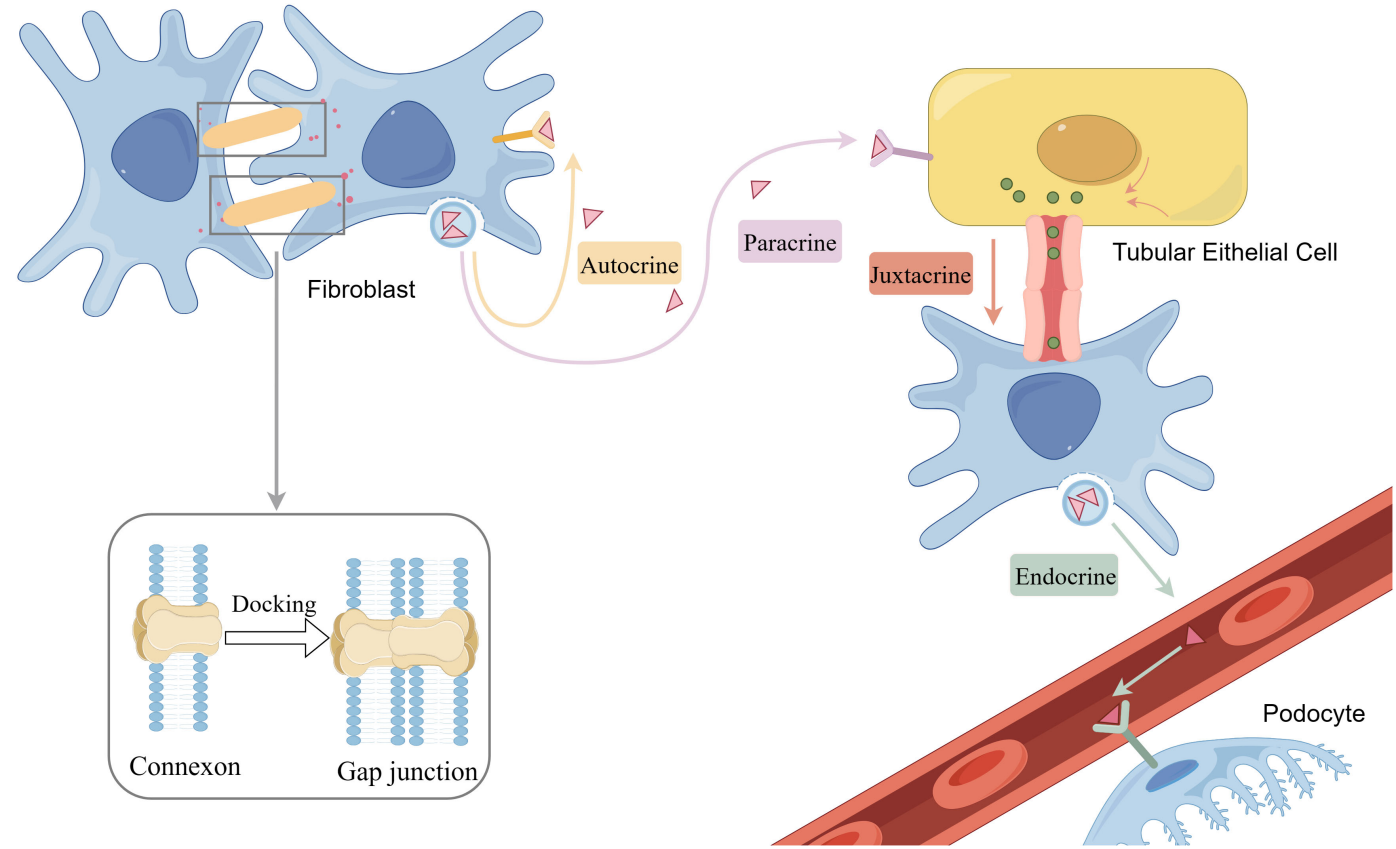
Gap junction and chemical communication including paracrine, autocrine, endocrine and juxtacrine. By figdraw.

Direct cell-to-cell communication is essential for maintaining the normal function of tissues and organs. Through these modes of communication, cells can effectively coordinate their activities in physiological processes and uphold overall homeostasis. Traditionally, direct communication between cells is considered to include gap junctions and juxtacrine interactions. Recently, tunneling nanotubes have also been recognized as a newer form of direct communication. (**Figure 2**) In DN, disruptions in direct communication can lead to pathological changes, and its dysregulation in pathological states may further exacerbate the progression of DN. Therefore, it is crucial to investigate the role of direct communication in DN. Because the role of juxtacrine interactions in DN has not yet been studied, following part will explore recent advancements in our understanding of gap junctions, tunnel nanotubes in DN, and insights from single-cell sequencing.

### 2.1 Gap junction

Gap junctions are specialized regions of the plasma membrane that contain arrays of intercellular channels, facilitating direct communication between the cytoplasm of neighboring cells. These channels enable the transfer of small molecules, ions, and signaling molecules, allowing for coordinated and synchronized physiological activities among adjacent cells, essential for normal tissue and organ function. The primary components of gap junctions are connexins, a class of transmembrane proteins that form the basic units of these channels (14). Humans possess a variety of connexins, including connexin 43 (Cx43), connexin 26 (Cx26), and others. Connexins assemble into hemichannels, comprised of six membrane-spanning helical structures. The hemichannels of neighboring cells can align and connect through a series of proteins, forming complete channels known as gap junctions (15). Hemichannels can be composed of one or more Cx isoforms (16). Once synthesized and inserted into the cell membrane, hemichannels composed of similar or different Cx isoforms can dock with each other to form homotypic or heterotypic gap junctions. Each Cx isoform has unique properties in terms of permeability, selectivity, conductance, and gating, which means that the numerous possible combinations can lead to a remarkable diversity of channel functions (17).

The role of gap junctions as a primary mode of direct communication has been increasingly recognized and applied in the context of DN. Changes in connexin expression and function have been linked to the initiation and progression of secondary microvascular complications associated with diabetes. Glucose has been shown to reduce gap junction conductance and disrupt cellular homeostasis in various cell types (18–20). Hills et al. summarized that the role of connexin-mediated intercellular communication is altered early in DN and may serve as an early recognition of DN. Based on studies of the kidney, nine connexin Cxs (Cx26, -30, -31, -32, -37, -40, -43, -45, and -46) have been recognized to be expressed in the kidney. Research teams have localized various connexins in the kidney. Cx26 mRNA is strongly expressed in the proximal tubules, Cx30 mRNA is selectively detected in urothelial cells, Cx32 mRNA is found in the proximal tubules and to a lesser extent in the collecting ducts. Cx37 mRNA is primarily associated with vascular endothelial cells, Cx40 mRNA is mainly present in glomerular mesangial cells and less so in vascular endothelial cells, Cx43 mRNA is expressed in small amounts in stromal cells across all kidney regions, and Cx45 mRNA is mainly found in the smooth muscle cell layers of blood vessels and ureters, as well as in mesangial and stromal (fibroblast) cells. Cx46 mRNA could not be detected (21). Among these, the connexins associated with DN include Cx43, Cx32, and Cx40. The roles of these three connexins in DN will be discussed below.

Connexin 43 (Cx43) is one of the most well-studied and ubiquitously expressed connexin isoforms in human tissues (22). The Hllis team first demonstrated that the expression of Cx43 is reduced in mesangial and extraglomerular cells in diabetic rats (23). They later confirmed that this protein is involved in the development and progression of DN. In diabetic conditions, reduced expression of Cx43 in rats induces senescence in glomerular mesangial cells (24). According to Sawai et al. (25), biopsies from diabetic patients show decreased Cx43 in podocytes. The progression of DN and the level of renal failure are associated with lower levels of Cx43 in podocytes. Additionally, high glucose-mediated reduction in Cx43 expression and gap junction intercellular communication activity contributes to the hypertrophy and senescence of glomerular mesangial cells (26). Consequently, Cx43 is now utilized as a marker to assess DN progression in humans.

Recent research has also begun to uncover the signaling pathways upstream and downstream of Cx43. In 2021, Kato et al. suggested that the miR-30s/Cx43/ERS axis could be a potential new target for DN therapy (27). Cx43 plays a crucial role in regulating renal epithelial-mesenchymal transition (EMT) and diabetic RF by modulating the SIRT1-HIF-1α signaling pathway. This provides experimental evidence for considering Cx43 as a potential target for DN (28). Despite these advancements, the clinical application of targeting connexins to manipulate cell-to-cell communication and influence DN development remains limited. Continued research is needed to explore how this critical issue can be effectively applied in clinical therapy.

It is noteworthy that the Hills team observed increased expression of Cx43 in the tubular regions of kidney biopsies from DN patients (29). In human collecting duct cell lines, there is a linear correlation between glucose concentration and Cx43 levels. Elevated glucose levels induce an increase in Cx43, facilitating cell communication via gap junctions (30). This finding appears to contradict the previous research. However, subsequent studies have clarified this discrepancy by showing that, in addition to their role in forming gap junctions, connexin hemichannels can independently influence intercellular communication (29). Chronic exposure to glucose-induced TGFβ1 has been shown to induce an increase in Cx43 expression, as suggested in the literature. Despite the increase in connexin expression, direct gap junction-mediated intercellular communication is reduced. In contrast, hemichannel expression and/or function, as well as ATP paracrine release, are increased. These changes trigger an increase in interleukin-6 (IL-6) and fibronectin expression levels. Additionally, the literature demonstrates increased expression of inflammatory and pro-fibrotic markers in ATP-treated human primary proximal tubule cells (31, 32). Elevated ATP levels have previously been linked to inflammation and fibrosis in various disease states. On this basis, the team further proposed that elevated TGF-β1 levels increase Cx43 hemichannel-mediated ATP release, an effect that drives purinergic receptor P2X7-mediated phenotypic changes, thereby triggering epithelial-mesenchymal-transformation in renal tubular regions (33). As early as 2013, studies have been devoted to the application of alkaloids to ameliorate the effects of high-glucose environments on hemichannels (34). In thylakoid cells, Valsartan prevented increased oxidative stress, decreased gap junction communication, and increased cell permeability due to high glucose and pro-inflammatory cytokine-induced connexin hemichannel activity. Other research indicates that, *in vitro*, Tonabersat inhibits the glucose/cytokine-dependent increase in ATP release mediated by Cx43 hemichannels. Additionally, Tonabersat reduces the expression of inflammation markers and the activation of the NLRP3 inflammasome in renal proximal tubular epithelial cells (35). However, it lacks clinical support and its applicability in human DN remains to be determined.

In addition to Cx43, other connexins associated with DN include Cx40 and Cx32. Research has shown that diabetes significantly reduces the expression of Cx40 in the renal cortex of rats (36). High glucose treatment leads to decreased expression of Cx40 in glomerular endothelial cells, though the underlying mechanisms remain to be further explored.Cx32 also exhibits a downregulation trend in DN. One study demonstrated that overexpression of Cx32 improves RF in diabetic mice by promoting K48-linked NADPH oxidase 4 (Nox4) polyubiquitination and degradation, thereby alleviating kidney fibrosis (37). Additionally, another study confirmed that overexpression of Cx32 significantly reduces reactive oxygen species (ROS) production and effectively inhibits the excessive production of extracellular matrix components, such as fibronectin (FN) and intercellular adhesion molecule-1 (ICAM-1), in high glucose-induced glomerular mesangial cells (38). This overexpression activates the Sirt1/Foxo3a pathway and suppresses oxidative stress in renal tissues, ultimately improving kidney function and glomerulosclerosis in diabetic mice. Recent advancements in therapeutic applications have emerged in 2020, where resveratrol has been shown to restore Cx32 expression, promote K48-linked polyubiquitination, and enhance Nox4 degradation, thereby reducing renal oxidative stress and improving the pathological progression of diabetes (37).

Overall, connexins play diverse roles in DN, and their therapeutic potential remains promising and warrants further exploration and research.

### 2.2 Tunnel nanotubes

TNTs are elongated membrane structures, typically between 50 and 200 nanometers in diameter and up to a few micrometers in length, that connect neighboring cells, enabling direct cell-to-cell communication (39). TNTs are unique cell protrusions whose formation mechanisms are not yet fully understood, but two primary views exist (40): filopodia interaction and cell shedding. In the filopodia interaction model, a filamentous pseudopod protrudes from one cell and elongates until it encounters another cell. In the cell-shedding model, cells initially in contact separate but remain connected through membrane structures. Both mechanisms may coexist (41–43).

TNTs play crucial roles in intercellular communication through several mechanisms:1)Transmission of substances: TNTs allow the direct transfer of various molecules and organelles, such as proteins, lipids, small molecules, RNAs, and mitochondria, between cells. This transmission helps maintain cellular functions and metabolic activities. 2)Signaling: TNTs transmit cellular signaling molecules (e.g., calcium ions, signaling proteins), enabling signaling communication between cells and regulating cellular physiological states and responses. 3)Electrical signaling: TNTs transmit electrical signals, synchronizing the electrical activities of cells. In neuronal cells, TNTs may facilitate the rapid propagation of electrical signals. 4) Immune response: TNTs enhance the efficiency of the immune response by transmitting information and facilitating cooperation between immune cells. For example, dendritic cells and macrophages use TNTs to transmit antigenic and immune signals, enhancing the immune response (44, 45).

Regarding diabetic nephropathy (DN), few studies have explored TNTs (46), but their findings are valuable. F. Barutta et al. demonstrated that mouse podocytes lacking the tumor necrosis factor-inducible protein 2 (TNFAIP2)-TNT system are more sensitive to the deleterious effects of diabetes, as evidenced by autophagosome accumulation and lysosomal dysfunction. *In vitro*, treatment with glycosylation end product-bovine serum albumin and high glucose increased the expression of microtubule-associated protein 1 light chain 3-II and autophagy receptor proteins. TNFAIP2 knockdown further exacerbated this effect, suggesting a reduction in autophagic flux. TNTs facilitated the translocation of healthy lysosomes from healthy to damaged podocytes, thereby restoring lysosomal function. Bidirectional transfer of autophagosomes was also observed, potentially alleviating the overload of damaged autophagosomes.

This study reveals that TNTs play a key role in regulating podocyte autophagy and maintaining cellular health in DN by mediating intercellular lysosomal and autophagosomal trafficking via a TNFAIP2-dependent mechanism. Although existing studies suggest that TNTs play an important role in DN, their specific mechanisms require further in-depth study. The current understanding of the molecular mechanisms and regulatory pathways of TNTs in intercellular communication is still limited, necessitating more experimental validation and exploration. Additionally, the study focused on *in vitro* experiments, lacking sufficient *in vivo* data to support the specific roles and mechanisms of TNTs. Therefore, further validation of TNT functions and their potential therapeutic effects in DN in animal models and clinical samples is needed. While TNTs have shown promise in basic research, translating these findings into clinical therapeutics faces challenges, such as effectively modulating TNT functions in patients and ensuring treatment specificity and safety. These issues need further investigation and resolution.

## 3 Indirect communication

### 3.1 Chemical communication

#### 3.1.1 The ways and effects of ligand-receptor binding on DN

Indirect communication includes chemical communication and extracellular vesicles. Chemical communication plays a role in DN specifically by regulating intracellular and intercellular signaling, influencing physiological and pathological processes in renal tissues. This includes various communication pathways such as autocrine, endocrine, and paracrine signaling, the dysregulation of which can lead to inflammation, cellular damage, and structural alterations in the kidneys, exacerbating the progression of DN.

The involvement of autocrine and paracrine mediators, including cytokines, prostaglandins, and leukotrienes, in triggering and maintaining inflammation in DN is well established (47). Additionally, it has been suggested that glomerular endothelial cell activity and angiogenesis are regulated by podocytes, tethered cells, glomerular endothelial cells, and immune cells in a paracrine and/or autocrine manner (48). Similarly, in other pathophysiological processes in DN, such as the pruning of the renal microvasculature, chemical communication through juxtacrine endothelial signals or paracrine signals from adjacent cells plays a crucial role in RF progression (49).

Studies on the mechanisms underlying these processes are emerging. For example, Quan Hong et al. proposed that TGF-β-1-mediated Smad3 activation in renal fibroblasts enhances LRG1 (leucine-rich α-2 glycoprotein-1) expression, leading to epithelial injury and amplification of TGF-β signaling through autocrine and paracrine modalities, promoting renal tubulointerstitial fibrosis in DN (50). Delma Vero et al. found that Vascular Endothelial Growth Factor A(VEGF-A) signaling in podocytes is mediated through the autocrine and paracrine pathways of Vascular Endothelial Growth Factor-2(VEGF2), which may mediate VEGF164 overexpression, triggering aberrations in the glomerular filtration barrier structure and function (51). BMP7, a podocyte differentiation and survival factor, and its signaling protein Smad5 function in an autocrine, paracrine, and/or, endocrine manner, and their reduction may lead to podocyte injury and loss (52). Notch signaling promotes overexpression in renal tubular epithelial cells, while cellular dedifferentiation and fibroblast activation occur through proximal secretory signaling (53).

Additionally, the adipocytokine Apelin has been shown to induce endothelial cell outgrowth by autocrine or paracrine means, contributing to neovascularization (54), which is implicated in the pathogenesis of DN (55). Therapeutic applications targeting these mechanisms are an important focus of current medical research. For instance, endothelin, which is linked to renal pathophysiology, exerts its effects through autocrine, paracrine, and endocrine signaling pathways, regulating aldosterone, catecholamines, and angiotensin, and antagonizing endothelin has been shown to reduce DN proteinuria. Studies have also suggested that Tilia tridentata extract and its major constituents hold promise as potential plants for preventing diabetic organ complications and damage by improving the endogenous antioxidant system and ameliorating inflammatory and endocrine dysregulation (56). However, translating these mechanisms for clinical application requires further exploration.

It is important to note that DN can also cause lesions in other organs, such as the heart, through endocrine mechanisms after developing into CKD (57). In conclusion, cell-cell interactions mediated by growth factors, cytokines, and chemokines occur in an autocrine, paracrine, endocrine, and juxtacrine manner, highlighting the complex interplay of chemical communication in the pathogenesis and progression of DN (58, 59).

#### 3.1.2 Single-cell sequencing insights into ligand-receptor binding

The intricate and diverse nature of renal structure and function in both healthy and diseased states presents a significant challenge in comprehending the mechanisms underlying the onset and progression of DN. Paracrine, autocrine, and endocrine modes of chemical communication play complex roles in DN, all mediated through signaling via ligand-receptor interactions.

Single-cell transcriptomics emerges as an ideal method for studying specific cell types and states, including the expression of genes or pathways, cellular differentiation trajectories, and gene regulation or co-expression in DN (60). **Table 1** (61–69) provides a summary of the results from single-cell RNA sequencing applied in DN, highlighting the mechanisms elucidated through this approach. **Table 2** (70–80) presents a re-analysis of DN single-cell RNA sequencing datasets from previous studies, showcasing the results and important findings.

**Table 1:** A summary of the results from single-cell RNA sequencing applied in DN.

Article Information	The Year of Publication	Disease/Model	Cell Number	Tissue type	Single-cell technique	Key Results
41	2022	Diabetic mouse models	63209	Kidney	10×Genomics Chromium	Textural variations in podocyte nuclei may be key to understanding the pathophysiology behind podocyte injury.
42	2022	5/6 nephrectomies rats	89420	Kidney	Illumina Hiseq X	The beneficial effects of empagliflozin on kidney function and morphology in 5/6 nephrectomyiced rats with established CKD are at least partially due to an inhibition of CD206 +CD68+ M2.
43	2023	db/db and db/m mice	42,833	Kidney	10× Genomics Chromium and Illumina	Crosstalk between podocytes and tubular cells in the proximal tubules was enhanced, and renal inflammation, oxidative stress, and fibrosis pathways were activated in db/db mice.
44	2023	Diabetic ZSF1 rats	4,821	Kidney	10x Genomics	Diabetic ZSF1 rats recapitulated functional and renal histopathological changes of human DKD. Pharmacological sGCact ameliorated functional and histological changes of DKD, while sGCstim had modest effects on ZSF rats.
45	2019	STZ-diabetic eNOS^−/−^ mice	829	Glomerular Cells	Fluidigm C1 & Illumina NextSeq 500	Demonstrate the ability of scRNA-seq analysis in isolated glomerular cells from diabetic and control mice to reveal dynamic changes in gene expression in diabetic kidneys, with variable responses of individual cells.
46	2022	db/db mice	83,585	Kidney	10×Genomics Chromium and Illumina NovaSeq 6,000	ARBs had more anti-inflammatory and anti-fibrotic effects, while SGLT2i affected more mitochondrial function in proximal tubular cells.The study also identified a new PT subcluster, was increased in DKD, but reversed by the treatments.
47	2019	Early DKD	23980	Kidney	10×Genomics Chromium & snRNA-seq	Increased potassium secretion and angiogenic signaling represent early kidney responses in human diabetic nephropathy.
48	2023	db/m mice and db/db mice	6775	Kidney	\	Identified the hub genes participating in the pathophysiology of early DKD and cross-talk of signaling transmission in early DKD.
49	2023	Human DKD kidney	62063	Kidney	10×Genomics Chromium	Found that renal inflammation involving upregulated inflammatory signaling pathways, release of chemokines and immune cell infiltration contribute to the pathogenesis and progression of DKD, especially in renal fibrosis.

**Table 2:** Re-analysis of DN single-cell RNA sequencing datasets from previous studies.

Article Information	The Year of Publication	Database	Dataset	Application	Conclusion
50	2020	the Gene Expression Omnibus	GSE131882	select 2000 genes to enrich with other datasets validate 133 differentially expressed genes	EGF, KNG1, GADD45B, and CDH2 might have reno-protective roles in DKD. Meanwhile, ATF3, B2M, VCAM1, CLDN4, SPP1, SOX9, JAG1, C3, and CD24 might promote the progression of DKD.
51	2021	the Gene Expression Omnibus	GSE131882	analysis and validate the expression of C7 with other datasets	ScRNA-seq analysis revealed that C7 was specifically highly expressed in the mesangial cells in DN.C7 is a potential MES cell-specific biomarker of DN.
52	2023	the Gene Expression Omnibus	GSE131882	analyze the immune microenvironment of DN identify 18 cell clusters with other datasets	LCK, CD3D, TLR7, and IL7R was considered to be the four most important genes for diagnosis by the mean decrease in the Gini index and a high portion of immune cell clusters (B cells, monocytes, and natural killer T cells) was detected in DN samples.
53	2023	the Gene Expression Omnibus	GSE165267	plot Uniform Manifold Approximation and Projection compute the differential gene expression in the comparison of Early db/db vs. Control and Late db/db	compared to the control group, adiponectin receptor levels decreased.This reduction in adiponectin receptor levels was associated with an upregulation of apoptosis-related genes, specifically Casp3 and Casp9, and a downregulation of antioxidant genes such as Gpx4, Gpx1, and Sod1.
54	2023	the Gene Expression Omnibus	GSE131882	validate other results and indicate that MYO1C and SP100 mRNA were expressed in various cell types	reported the whole-transcriptome genetic resources found in urine extracellular vesicles of T2DN patients
55	2023	the Gene Expression Omnibus	GSE131882	resolved cellular heterogeneity and screened key genes with DEGs and WGCNA	The SLIT3、PDE1A and CFH immune-associated genes could be used as diagnostic markers and therapeutic targets of DKD.
56	2023	the Gene Expression Omnibus	GSE131882、GSE195797、GSE212273	plot Uniform Manifold Approximation and Projection、evaluate the expression of the genes	Podocytes express low amounts of the NLRP3 inflammasome, if at all, and do not produce IL-1b and IL-18, not even upon introduction of the A350V Muckle-Wells NLRP3 variant and upon induction of podocyte stress. NLRP3mediated glomerular inflammation is limited to immune cells
57	2023	the Gene Expression Omnibus	GSE131882、GSE195460、GSE151302、GSE195460、 GSE131685	Identify cell types、plot Uniform Manifold Approximation and Projection、 screened key genes	The interaction between macrophage efferocytosis and intrinsic kidney cells was enriched in the regulation of the MAPK pathway, immune system, RAF/MAP kinase cascade, cytokine signaling immune system, and costimulation by the CD28 family.RAC1 facilitates macrophage efferocytosis
58	2023	the Gene Expression Omnibus	GSE127235	select highly variable genes、plot Uniform Manifold Approximation and Projection	SYK, ITGB2, FCER1G, and VAV1 were identified as immunological markers of DKD with promising predictive ability.
59	2022	the Gene Expression Omnibus	GSE131882	compare the hallmark enrichment profiles with a glomerular dataset 、provide information to identify cell-type-specific responses in bulk data	Identified TEKT2 and PIAS2, two spermatogenesis-related genes involved in the pathogenesis of DN. Furthermore, TEKT2 is involved in this pathogenesis by regulating the podocyte cytoskeleton.
60	2023	the Gene Expression Omnibus	GSE127235	Identify cell types、plot Uniform Manifold Approximation and Projection、 screened key genes	FOS and ZFP36 may play an anti-aging role in DN to ameliorate cell intracellular premature aging in mesangial cells of glomeruli.

Taken together, single-cell sequencing technology offers a potent tool for understanding the mechanisms of intercellular communication during DN development, identifying potential therapeutic targets, and discovering biomarkers for clinical applications.

### 3.2 Extracellular vesicles

EVs are key players in intercellular communication and can be classified into exosomes, microvesicles, apoptotic vesicles, and FTVs. Studies indicate that EVs mediate intercellular communication in DN development (91). Notably, FTVs represent a special form of EVs (92). This article categorizes EVs, focusing on the roles of exosomes and FTVs in DN.

#### 3.2.1 Exosomes

**Figure 3:**
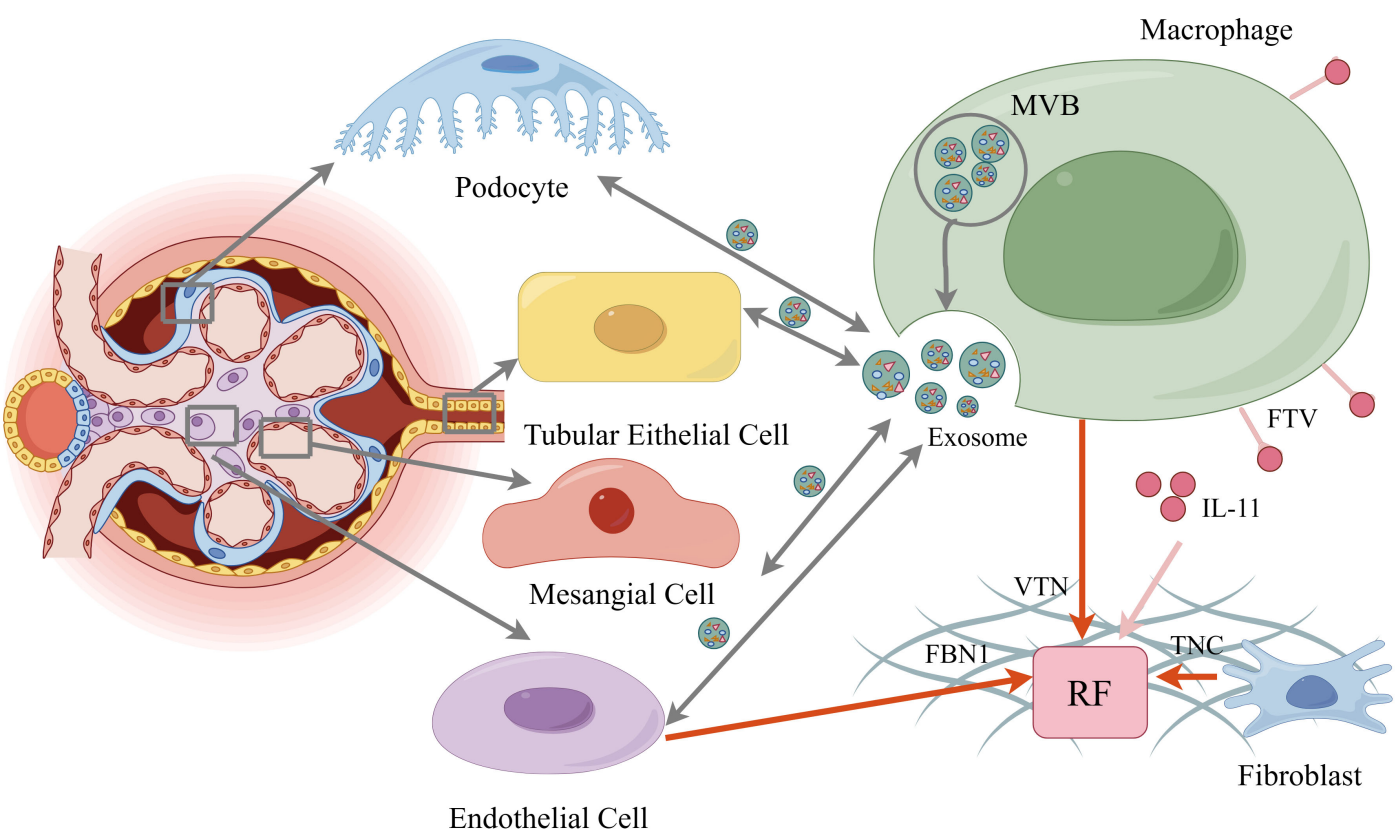
The various cells communicate by secreting exosomes. Macrophage also release FTV from their elongated filamentous pseudopods, which include IL-11, leading to renal fibrosis. In addition, extracellular matrix glycoproteins, FBN1, VTN, TNC from endothelial cell, macrophage, fibroblast constitute the crucial part of fibrogenic niche. By figdraw.

In recent years, exosomes have garnered significant research attention as crucial mediators of intercellular communication. These EVs, originating from endosomes, typically range in diameter from 40 to 160 nm. Exosomes carry a diverse array of biomolecules, including proteins, lipids, and nucleic acids, among others (93). They are capable of transmitting information in extracellular fluid through their cargo, influencing the physiological state of recipient cells (**Figure 3**). Exosomes play pivotal roles in various physiological and pathological processes, including immune regulation and disease development (94, 95).

Recent studies have increasingly highlighted the role of exosomes in disease processes, with DN emerging as a particularly intriguing area of investigation. Exosomes play a crucial role in regulating renal inflammatory responses (96),mediating EMT (97), and reducing oxidative stress (98) through intercellular communication. Research has shown that glucose levels can influence the secretion and content of exosomes from human thylakoid cells, further implicating exosomes in DN pathogenesis (99). Analysis of DN datasets has revealed that exosomes are enriched for differential genes expressed in DN, underscoring their importance in the disease (100, 101). While this article does not delve into the details of exosome biosynthesis and release, as these have been extensively covered in a review by Kalluri et al., it focuses on the role of exosomes in DN, particularly in intercellular communication during disease progression (93). Recent advances in understanding exosome-mediated communication between different cell types in DN are emphasized, highlighting the evolving landscape of DN research.

##### 3.2.1.1 Exosome-mediated intercellular communication within the nephron

In the nephron, each type of cell has a specific role, and damage to any cell type in a high glucose environment can lead to irreversible nephron damage. The destruction of podocytes and endothelial cells is closely associated with the early development of proteinuria in DN. The reduction in podocyte numbers and detachment from the glomerular basement membrane compromise the integrity of the glomerular filtration barrier, leading to proteinuria and accelerating podocyte loss. Animal studies have shown that more than 20% podocyte loss results in irreversible glomerular damage, ultimately leading to end-stage renal disease (102). Changes in podocyte phenotype, damage to slit diaphragm structure and function, and podocyte detachment are major contributors to proteinuria and DN progression (103). Additionally, hyperglycemia-induced oxidative stress and upregulation of pro-inflammatory factors like TGF-β1 promote mesangial cell proliferation and mesangial matrix accumulation (104).

Within the nephron, exosomes are involved in mediating intercellular communication, particularly through proximal-distal signaling (105). Podocytes play a critical role in this process. For instance, exosomes derived from podocytes in a high glucose environment can induce dedifferentiation of proximal tubular epithelial cells (81). Additionally, exosomes from podocytes in DN are key mediators of proximal tubular cell injury, potentially through miR-221-mediated Wnt/β-catenin signaling (106). These findings suggest that exosomes facilitate proximal-distal signaling within the nephron, and disrupting this process might offer new strategies for treating DN.

Research also shows that exosomes can have protective effects against podocyte injury. For example, miR-16-5p from urinary exosomes can protect against podocyte damage induced by high glucose and inhibit VEGFA (Vascular Endothelial Growth Factor A) expression and podocyte apoptosis (82). Similarly, exosomes derived from adipose-derived stem cells can significantly improve DN symptoms by enhancing miR-486 expression and inhibiting the Smad1/mTOR signaling pathway in podocytes (83). Other studies have indicated that miR-22-3p in exosomes protects podocytes and diabetic mice from inflammation by inhibiting NLRP3 inflammasome activation (84). These results underscore the potential of exosomes as mediators of intercellular communication that can mitigate podocyte injury and improve renal function recovery. Exploring the use of exosomes to protect various cell types within the nephron represents a promising avenue for research and therapeutic development.

##### 3.2.1.2 Exosome-mediated communication between nephron cells and other cells

RF and inflammation play central roles in the onset and progression of DN. In the diabetic microenvironment, elevated glucose levels, reactive oxygen species (ROS), and oxidative stress contribute to renal cell injury and the release of injury factors, triggering pro-inflammatory signals. This continuous immune system activation results in the production of inflammatory mediators within renal cells, leading to the recruitment and activation of macrophages and monocytes, further amplifying the inflammatory response (107, 108). Prolonged chronic inflammation ultimately leads to kidney injury in DN. Infiltration of inflammatory cells is also an initiating factor in RF. These cells secrete various inflammatory cytokines, with TGF-β1 being closely associated with RF (109). During the early stages of DN, TGF-β1, a key inflammatory cytokine in RF, activates downstream signaling pathways such as Smad2/3, MAPKs, PI3K/AKT, and Wnt/β-catenin. This activation promotes the synthesis of ECM and the transformation of fibroblasts into myofibroblasts, accelerating the progression of RF (110). Myofibroblast transdifferentiation and EMT also contribute to RF (109). Research indicates that macrophages play a crucial role in cell-to-cell communication.

Macrophages, as important inflammatory cells, can both trigger inflammatory responses and be regulated by inflammatory factors. One of the mediators of communication between macrophages, inflammatory factors, and resident kidney cells is exosomes. Exosomes mediate communication between macrophages and mesangial cells by activating the TGF-β1/Smad3 signaling pathway in a high-glucose environment and exert effects through the inflammasome (NLRP3) (88). NLRP3 is an immune protein that, when activated, forms a complex with other proteins known as the NLRP3 inflammasome. This complex releases pro-inflammatory factors such as interleukin-1β (IL-1β) and interleukin-18 (IL-18), thereby triggering an inflammatory response.

However, the relationship between exosomes and macrophages goes beyond this. Under different stimulus environments, macrophages can be activated into different phenotypes: M1 macrophages and their exosomes are involved in the early stages of inflammation, associated with tissue damage and pro-inflammatory functions, while M2 macrophages and their exosomes release cytokines that alleviate inflammation and exert anti-inflammatory effects (111).

Lin-Li Lv and colleagues proposed (85) that exosomal miR-19b-3p mediates communication between injured tubular epithelial cells and macrophages, leading to M1 macrophage activation. They also revealed the mechanism whereby tubular epithelial cell-derived exosomal miR-19b-3p is internalized by macrophages, leading to M1 phenotype polarization by targeting NF-κB/Suppressor of cytokine signaling-1 (SOCS-1). NF-κB is a classic signaling pathway in DN involving inflammation and RF, and SOCS-1 is a protein that can negatively regulate cytokine signaling. Similarly, another study indicated that high glucose environments promote M1 macrophage polarization and the production of more exosomes, thereby activating mesangial cells and increasing exosome internalization (112). Yi-Shen-Hua-Shi granules might alleviate podocyte injury by inhibiting M1 macrophage polarization and reducing macrophage-derived exosomal miR-21a-5p, thereby interfering with DN progression (113).

Exosomes secreted by M2 macrophages attenuate lipopolysaccharide-induced podocyte apoptosis through the miR-93-5p/TLR4 axis (86). This type of macrophage can also secrete exosomal miR-25-3p, which activates autophagy by inhibiting DUSP1 expression, thus improving high glucose-induced podocyte injury (87).

##### 3.2.1.3 Cell death

Exosomes play a multifaceted role not only in normal and pathological cellular processes but also in cell death and senescence. Notably, certain exosomes can mitigate podocyte apoptosis, ameliorating proteinuria symptoms (82, 114). For instance, exosomes derived from bone marrow mesenchymal stem cells containing miR-30e-5p inhibit caspase-1-mediated thermoapoptosis induced by high glucose in HK-2 cells by targeting ELAVL1 (89). On the other hand, miR-4449 in serum exosomes promotes the expression of pro-inflammatory genes, elevates reactive oxygen species levels, and impacts cellular focalization. Interestingly, the effects of miR-4449 can be counteracted by N-acetylcysteine (NAC), a common dietary supplement (90). The non-coding RNAs and mechanism mentioned above are summarized in **Table 3**.

**Table 3:** The summary of recent advances in the role of non-coding RNAs in exosomes in diabetic nephropathy.

noncoding RNA	Mechanism	Origin of exosomes	Target Cell	Reference
miR-221	mediate the dedifferentiation of renal proximal tubule cells	podocytes	renal proximal tubule cells	(81)
miR-16-5p	suppress VEGFA expression and podocytic apoptosis	urine-derived stem cells	podocytes	(82)
miR-486	inhibit Smad1/mTOR signaling pathway	adipose-derived stem cells	podocytes	(83)
miR-22-3p	inhibit the NLRP3 Signaling Pathway	Mesenchymal Stem Cell-Derived	podocytes	(84)
miR-19b-3p	lead to M1 macrophage polarization through targeting NF-κB/SOCS-1	tubular epithelial cells	M1 macrophage	(85)
miRNA-93-5p	improve lipopolysaccharide-induced podocyte apoptosis by targeting Toll-like receptor 4	M2 macrophages	podocytes	(86)
miR-25-3p	activation autophagy via inhibiting DUSP1 expression	M2 macrophages	podocytes	(87)
lncRNA Nuclear-enriched transcription-1	target microRNA to promote extracellular matrix accumulation and EMT via the Akt/mTOR signaling pathway	macrophages	renal tubular epithelial cells	(88)
miR-30e-5p	induce renal proximal tubular cell pyroptosis by inhibiting ELAVL1	Bone marrow mesenchymal stem cell	renal proximal tubular cell	(89)
miR-4449	promote pyroptosis and oxidative stress through the miR-4449/HIC1 pathway	Serum	renal tubular epithelial cells	(90)

The utilization of exosomes and their contents presents a significant advantage as a biomarker for the early detection of DN. Traditionally, proteinuria has been considered a biomarker, but its limitation lies in its late onset, indicating an advanced stage of DN once microalbuminuria is detected (115).Therefore, there is a need for new early markers for timely detection and treatment. Extensive research is exploring the potential role of exosomes. The use of exosomes as biomarkers involves a series of detailed steps. Firstly, samples need to be collected from biological specimens such as blood, urine, or saliva. It is crucial to adhere to standard operating procedures to maintain sample quality and exosome integrity. After sample collection, the next step is exosome isolation. Common methods include ultracentrifugation, which involves a multi-step centrifugation process to remove cell debris and particles from the sample, ultimately collecting the exosomes. Another method is ultrafiltration, which separates exosomes based on molecular weight using filtration membranes. Additionally, immunoaffinity capture can be used, which involves isolating exosomes using antibodies that bind to specific antigens on the exosome surface, or size exclusion chromatography to separate exosomes based on particle size. Once exosomes are isolated, the next step is purification. Typically, ultrafiltration or size exclusion chromatography is used to further purify the exosomes and remove non-exosomal particles and other contaminants. Following purification, exosome characterization is performed. Nanoparticle tracking analysis is used to measure the size and concentration of exosomes, and transmission electron microscopy can be used to observe the morphology and structure of exosomes. Additionally, Western blot, ELISA, and other methods are used to detect characteristic surface proteins of exosomes (such as CD63, CD81, TSG101, etc.) for identification. Next is the analysis of exosome contents. RNA can be extracted from exosomes for miRNA and mRNA analysis, or proteins can be extracted for proteomics analysis using techniques such as mass spectrometry. Metabolomics analysis can also be performed to understand the metabolic profile of exosome contents. The subsequent step is biomarker identification. By correlating exosome content data with disease or physiological states, bioinformatics analysis is used to identify potential biomarkers. These biomarkers need to be validated in independent sample cohorts to assess their specificity and sensitivity. Such biomarkers can be applied in clinical settings, including early disease diagnosis and classification, prognosis assessment, and treatment monitoring. Ensuring that all steps are standardized is essential to ensure the reliability and reproducibility of exosomes as biomarkers in clinical applications (116–119).

However, the biological properties of exosomes are influenced by various factors, including cell type, state, and stimulation of the source, suggesting some degree of heterogeneity and instability (101, 120, 121). Extensive clinical validation is necessary to address this issue and enable the practical application of this new biomarker in clinical settings. It also have challenges in their translational process. One major hurdle is the complexity, technical difficulties, and high costs associated with exosome isolation and purification methods, which impede their transition from the laboratory to clinical settings. Although differential ultracentrifugation is the current gold standard for isolating exosomes, it is time-consuming (122). Immunoprecipitation and size filtration methods have been developed as alternatives but often result in extracellular vesicle mixture contamination. Addressing this challenge is a key area for researchers to explore further. Despite these challenges, exosomes remain a promising therapeutic target.

#### 3.2.2 Filopodial tip vesicles

We discussed the involvement of macrophages in the inflammatory and fibrotic processes of DN above. When recruited to the kidney under pathological conditions, fibroblasts can transdifferentiate into myofibroblasts, contributing to RF, a crucial process for restoring homeostasis after kidney injury (123). Targeting macrophage signaling pathways by depleting tissue macrophages and inhibiting their recruitment has emerged as a potential strategy to attenuate the progression of RF (124–126). Previous studies have highlighted the presence of specific filamentous pseudopods, such as cell lines, tunneling nanotubes, neutrophil trails, and migrants, in various tissues (127–129). These structures facilitate signaling between cells over different distances by transporting substances like mitochondria, endosomal vesicles, and pathway ligands in a highly specific manner (130). Recently, macrophage filamentous pseudopodia, which are specialized cellular protrusions primarily used for capturing and retrieving pathogens, have been observed engaging in this form of communication (131). For example, mouse peritoneal macrophages and RAW 264.7 cell macrophages release cholesterol-rich vesicle particles ranging from 20 to 100 nm from their elongated filamentous pseudopods, indicating their potential to deliver cellular signaling molecules and mediate inter-cellular communication (132, 133). Xiaodong Zhu et al. discovered that the tips of these pseudopods can release a unique membrane structure known as FTVs (**Figure 3**) (92). These FTVs contain multiple internal vesicles and cargos and are transported via nanotubes. The team classified FTVs as a distinct form of extracellular vesicles due to their large vesicular structure, resembling classical extracellular vesicles and cytokines that carry selective mediators to receptor cells (134, 135). FTVs can detach from the tips of filamentous pseudopods and deliver numerous molecular signaling molecules to fibroblasts, influencing their differentiation and functional state.

Macrophages from various sources, including mouse peritoneal macrophages, the RAW 264.7 cell line, and human peripheral blood mononuclear cells, were used in this study under different stimulation conditions, such as M1-type polarization, high glucose exposure, and low oxygen levels. These conditions were selected to induce filamentous pseudopod formation and the release of FTVs during the experiments. A wide array of techniques and methods, including light microscopy, electron microscopy, flow cytometry, mass spectrometry, protein blotting, enzyme-linked immunosorbent assay (ELISA), fluorescence *in situ* hybridization (FISH), fluorescence resonance energy transfer (FRET), and luciferase reporter genes, were employed to observe and analyze the morphology, structure, composition, and function of FTVs. It was observed that FTVs from M1-type macrophages and those stimulated with high glucose were enriched in the chemokine interleukin-11 (IL-11), which plays a crucial role in fibroblast transdifferentiation.

The research team utilized a mouse model of DN and mouse renal interstitial fibroblasts to mimic DN both *in vivo* and *in vitro*. In the *in vivo* experiments, the impact of FTVs on renal interstitial fibrosis was evaluated by injecting diabetic mice with HG/M1-ftv via the tail vein, along with a control group. Additionally, an FTV inhibitor or an FTV IL11-antibody was administered to validate the significance of the FTV IL11 pathway. In the *in vitro* experiments, the effects of FTV on fibroblasts were investigated through *in vitro* culture and co-culture experiments. IL11-neutralizing antibodies or IL11 receptor antagonists were employed to disrupt FTV IL11 signaling.

The experimental findings demonstrated that HG/M1-ftv induced renal interstitial fibrosis in diabetic mice. Conversely, inhibition of FTV or targeting of the FTV IL11 pathway attenuated renal interstitial fibrosis. These results suggest that the HG/M1-ftv IL11 pathway represents a novel mechanism of RF in DN.

This discovery reveals a novel mechanism by which extracellular vesicles influence fibrosis in DN through intercellular communication. While the role of macrophages in promoting inflammation is well-established, their mechanism in resisting RF has been less studied. This research represents significant progress in this area and provides new insights into utilizing FTV to treat DN. This avenue of research will be a crucial focus for our future scientific endeavors.

## 4 Fibrotic niche

Despite the intricate and interrelated mechanisms underlying DN, it is evident that RF is a pivotal aspect of the DN progression. RF serves as the ultimate common pathway for the transformation to end-stage renal disease during the exacerbation process not only of DN but also of all the initial causes that can lead to CKD. Therefore, based on current research, reversing the process of RF is a critical focus area that demands our attention.

Numerous studies have revealed that renal fibrotic lesions do not manifest uniformly throughout the renal parenchyma. Instead, they typically initiate at focal sites where mesenchymal fibroblasts become activated, proliferate, and generate substantial amounts of ECM components (136). Importantly, following kidney injury, ECM proteins undergo qualitative and quantitative changes that influence the activation and function of neighboring cells, thereby altering their phenotype and course. Researchers have long postulated the existence of a specialized microenvironment within the ECM that determines the site of fibroblast activation. In 2017, Haiyan Fu et al. introduced the term “fibrotic niche” to describe this microenvironment, concurrently identifying part of its specific components-Tenascin-C(TNC), an ECM glycoprotein (137). (**Figure 3**). The concept of the fibrotic ecotone has also been applied to liver and lung fibrosis, emphasizing the similarities in tissue fibrogenesis across different organs (138–140). Subsequently, the team further elaborated on the contents of the fibrotic niche, proposing that it is scaffolded by the ECM and encompasses renal resident cells (e.g., renal tubular cells, peritubular capillary endothelial cells, pericytes, mesenchymal fibroblasts, and dendritic cells), infiltrating inflammatory cells (e.g., neutrophils, T-cells, and macrophages), extracellular vesicles, secreted soluble factors, and a specialized ECM network. Among these components, the ECM is believed to act as an “anchor” that centrally regulates cell adhesion, intercellular communication, and cell behavior within a three-dimensional environment. Upon kidney damage, inflammatory cells, extracellular vesicles, and soluble factors are recruited from the periphery to mediate intercellular communication, leading to alterations in the renal microenvironment that drive disease progression. It is through this mechanism that the ECM confines RF to a specific region (141).

This study also observed that proteins within the ECM can be broadly classified into four main groups: matrix cellular proteins, ECM structural proteins, proteoglycans, and matrix-modifying proteins. Significantly, stromal cell proteins exhibited the most up-regulation in CKD renal tissue scaffolds, indicating their crucial role in the fibrous matrix. Matrix cytosolic proteins can influence cell-matrix interactions, thereby regulating cell growth, migration, differentiation, apoptosis, and other functions (142).These proteins are typically expressed at low levels in normal kidney tissues but are re-induced following tissue injury. They can undergo rapid turnover and play a regulatory role when bound to ECM structural proteins. Additionally, they contain structural motifs commonly found in other types of matrix proteins, including integrins, cell surface proteoglycans, Toll-like receptors (TLRs), growth factor receptors, and scavenger receptors (143). Among these matrix proteins, one of the most extensively studied types is TNC, as mentioned earlier.

Like other stromal cell proteins, TNC interacts with ECM structural proteins and cell-surface receptors, despite being a nonstructural protein. Haiyan Fu et al. discovered that TNC is upregulated early in renal injury within fibroblast-rich localized renal parenchyma, contributing to the formation of fibrotic niche scaffolded by the ECM (137). Moreover, depletion of TNC attenuates fibroblast expansion and RF *in vivo*, while *in vitro* studies showed that TNC promotes fibroblast proliferation. Thus, TNC plays a significant role in the fibrotic niche. Additionally, this study demonstrated through ex vivo and *in vitro* experiments that TNC triggers EMT via the integrin αvβ6/FAK/ERK-1/2 signaling cascade, leading to impaired renal tubular integrity. This unveils one of the mechanisms through which TNC contributes to RF. Furthermore, the study showed that urinary TNC levels correlate with the severity of RF, suggesting its potential as a biomarker. Similarly, Haili Zhu et al. demonstrated the importance of TNC and its potential as a biomarker, suggesting that blocking the TNC/αvβ6 integrin/FAK signaling cascade may be a novel therapeutic strategy for intervening in RF. Thus, TNC is emerging as a potential biomarker for early-stage RF and a target for developing new therapeutic approaches. However, its accuracy as a biomarker requires determination and clinical validation, necessitating more in-depth and comprehensive studies for therapeutic translation.

In addition to TNC, many proteins constituting fibrotic niche are present in the ECM, and their roles are being elucidated. For example, macrophages promote RF by assembling fibrotic niche enriched with Vtn, a hyaluronan-binding protein. Endothelial cell secrete fibrillin 1(FBN1) (**Figure 3**) (144, 145). It has also been shown that oxidative stress is a key factor in the fibrotic microenvironment (146).The study of the fibrotic ecotone has become an emerging research direction with significant application prospects and potential. However, these studies have some shortcomings. Firstly, RF is a critical stage in the progression of CKD, and various diseases progress to CKD; is the alteration of fibrotic niche the same in RF caused by different etiologies? Moreover, the key mechanisms involved have not been fully elucidated and could be extended laterally to studies of other organs. For instance, Alexander R. Pinto et al. discovered that the stromal cell protein Cartilage Intermediate Layer Protein (CILP), frequently upregulated during cardiac pathological remodeling, plays a crucial role in safeguarding against sustained activity of TGF-β1, a key signaling molecule in fibrosis, and myofibroblast differentiation (147).This protein is also expressed in the kidney, suggesting a potential new direction for mechanistic research. Finally, translating this concept and mechanism into new drug therapies for the benefit of DN patients worldwide presents a significant challenge.

## 5 Discussion

Intercellular communication is vital for exchanging information between cells through various signaling methods, maintaining the coordination and balance of physiological functions within an organism. However, in pathological states, intercellular communication plays a crucial role in disease development. Therefore, understanding its impact on DN is essential. This review summarizes recent research advances in intercellular communication in DN, focusing on its modes of action and describing the pathological mechanisms involved. Direct communication, such as through gap junctions and TNTs are discussed, along with indirect communication mechanisms involving chemical communication (autocrine, paracrine, proximate, endocrine),exosomes, FTV, the fibrogenic niche. The review highlights the role of indirect communication in DN, including the transfer of information via exosomes between immune cells and renal units, and the secretion of IL-11 and other mediators by FTVs to promote fibroblast transdifferentiation. Various types of proteins in the fibrogenic niche are also implicated in promoting RF in DN, representing a significant advancement in understanding DN pathophysiology.

Furthermore, we summarizes the role of single-cell sequencing in DN research. Despite its continued development, there is a need to enhance its strengths and address its weaknesses, such as through joint batch sequencing for improved interoperability, expanding sample sizes, enhancing measurement technology, and minimizing errors. Moving forward, utilizing intercellular communication comprehensively and multidirectionally for DN treatment is a promising research direction.

Although the mechanisms underlying DN have been extensively studied and elucidated, providing valuable insights into the nature of the disease, their clinical application remains somewhat limited. Factors that may interfere with these mechanisms, the impact of other pathways on downstream signaling molecules, and whether it is the studied mediators themselves or the cargos wrapped in these mediators that play a role, all require further research and investigation.

Moreover, some studies have been confined to common animal models, which limits their ability to replicate human DN. Many strains of mice that develop diabetes (e.g., through streptozotocin injections) do not exhibit the phenotypes observed in DN patients, such as thylakoid dilatation, glomerular basement membrane thickening, tubulointerstitial damage, and endothelial hyalinization. Therefore, constructing animal models that better mimic the pathological state of human DN is an important area for investigation.

It is important to acknowledge the challenges and limitations in translating these mechanisms into clinical applications. Technical and methodological challenges exist, including whether drugs or other treatments targeting these mechanisms will impact other pathogenic factors, whether the benefits of such treatments outweigh the risks in clinical use, and how to effectively translate emerging research into clinical practice. Researchers must deepen their understanding of these mechanisms and find more innovative and practical ways to translate scientific discoveries into clinical applications.

This review highlights the significant role of intercellular communication in the treatment and prevention of DN. It emphasizes the need for further research to elucidate the specific mechanisms of emerging intercellular communication modalities in the onset and progression of DN. This research aims to identify new interventions that could effectively prevent and treat renal damage in diabetic patients.
